# Multi-regional transcriptomic analysis reveals early nociceptive and neuroinflammatory alterations in APP/PS1 mice

**DOI:** 10.3389/fimmu.2026.1937738

**Published:** 2026-08-26

**Authors:** Zhutao Sheng, Fu Xu, Yuqing Yan, Ling Chen, Baomin Dou, Na Liu, Sicheng Zhou, Xiongwei Zhu, Yuan-Xiang Tao, Ying Xu

**Affiliations:** 1 Department of Anesthesiology, Rutgers, the State University of New Jersey, Newark, NJ, United States; 2 Department of Pharmaceutical Sciences, School of Pharmacy, University at Buffalo, The State University of New York, Buffalo, NY, United States; 3 Department of Pathology, Case Western Reserve University School of Medicine, Cleveland, OH, United States

**Keywords:** Alzheimer’s disease, amyloid plaques, memory impairment, neuroinflammation, nociceptive hypersensitivity

## Abstract

**Background:**

BackgroundAlzheimer's disease (AD) is characterized by progressive cognitive decline and neuropsychiatric symptoms, including chronic neuropathic pain. However, the molecular mechanisms linking pain sensitivity to early AD pathology remain poorly understood.

**Methods:**

In the present study, 4- to 5-month-old APP/PS1 mice were used to identify molecular signatures of the early pathological stage. Transcriptomic analysis, quantitative real-time RT-PCR (qRT-PCR), immunoblot, and ELISA analyses were performed to assess region-specific gene expression, neuroinflammation, and synaptic protein levels in pain-related regions.

**Results:**

Although memory behaviors remained unchanged in 4–5-month-old APP/PS1 mice, these mice showed an increase in whole-brain Aβ plaque burden. Transcriptomic analysis and qRT-PCR revealed distinct region-specific gene expression changes. Plin4 was downregulated in the DRG, spinal cord, thalamus, and hippocampus of APP/PS1 mice. *Rps3a3* and *Prnp* were significantly upregulated in both the spinal cord and thalamus, whereas *Hif3a* and *Serpina3f* were significantly upregulated in the thalamus and hippocampus. These gene alterations were closely involved in inflammatory responses and synaptic plasticity. Immunoblot and ELISA analyses in these pain-related regions demonstrated increased TNF-α and IL-1β levels, as well as reduced expression of synaptic proteins, indicating enhanced neuroinflammation and synaptic loss in the early stages of APP/PS1 mice.

**Conclusion:**

These findings support the presence of neuroinflammation and synaptic dysfunction in pain-processing circuits as early molecular events in APP/PS1 mice, suggesting that nociceptive alterations may represent an early feature of the pathological stage of AD.

## 1 Introduction

Alzheimer’s disease (AD) is recognized by progressive memory loss and neuropsychiatric symptoms, currently affecting over 50 million people worldwide, especially among elderly people over 65 years old (1). This number is projected to reach approximately 78 million by 2030 (2, 3). Evidence from multiple studies indicates that patients in the early stages of AD also frequently experience neurological and neuropsychiatric symptoms, such as abnormal movements, depression, anxiety, and chronic pain (2, 4, 5). Some neuropsychiatric symptoms in AD patients may be underestimated due to their reduced ability to express discomfort as compared to cognitively healthy individuals. Pain has been reported in a subset of patients with AD and related dementias (6, 7). Interestingly, recent large-scale clinical investigations showed that approximately 45.8% of Alzheimer’s disease patients reported chronic pain with an age range of 48–103 years (5, 6). As one of the most common pain disorders, migraine is closely involved in cognitive impairment and the risk of dementia (8). Studies from large-scale analyses indicate that migraine was a risk factor for vascular dementia (VaD) and AD-related dementia (ADRD) (9, 10). This evidence strongly suggests that chronic pain may be associated with the development of cognitive and memory deficits. Moreover, recent studies demonstrated that individuals with mild cognitive impairment (MCI) who also suffered from neuropsychiatric disorders, such as chronic pain, often progressed more rapidly to ADRD (11–14), further indicating a potential causal relationship between pain hypersensitivity and early-stage cognitive dysfunction. However, molecular mechanisms underlying this relationship remain to be fully elucidated.

Nociceptive dysregulation may share common pathological pathways with early-stage cognitive impairment in the development of AD. Neuroinflammatory processes, particularly the upregulation of pro-inflammatory mediators such as TNF-α and IL-1β in both the brain and dorsal root ganglion (DRG), are among the earliest pathological triggers of nociceptive hypersensitivity in age-related neurocognitive disorders such as mild cognitive impairment (15–17). These mediators may exacerbate pathological neuronal remodeling, leading to heightened pain sensitivity and further aggravation of ADRD pathology. Some cases of AD are inherited, with mutations frequently occurring in genes that regulate metabolism, oxidative stress, and immune-infiltration pathways (18). The thalamus and hippocampus are recognized as key processing centers in the regulation of cognitive and affective functions and exhibit abnormally increased activity in individuals with chronic pain (19–21). Dysregulation of these genes in the thalamus and hippocampus may underlie the increased risk of chronic pain associated with dementia. The spinal cord and DRG may serve as the key regions where nociceptive information is transmitted and modified. Dysregulation of neurotrophic signaling and synaptic remodeling of primary afferents in the DRG are key mechanisms underlying chronic pain (22). Aging further exacerbates both peripheral (DRG) and central (spinal cord, thalamus, and hippocampus) abnormalities associated with chronic pain (23–25).

Considering that late-stage AD patients exhibit reduced pain perception, mainly due to gray matter and memory loss (26, 27), the present study primarily focused on evaluating transcriptomic changes across anatomically distinct neural regions in an early stage of an AD mouse model. Through comprehensive behavioral assessments and RNA sequencing (RNA-seq) analyses, we identified distinct patterns of gene expression associated with neuroinflammatory and synaptic remodeling pathways. Six genes, *Plin4, Hif3a, Rps3a3, Serpina3f, Hmga1b, and Prnp*, were consistently dysregulated across both central and peripheral pain-related regions, including the thalamus, hippocampus, DRG, and spinal cord from the APP/PS1 mice. The functional gene ontology (GO) enrichment analysis showed that these genes were essential for modulating inflammatory responses and neuroimmune interactions. The multi-regional transcriptome maps in the present study provide new insights into the systemic nature of alterations in pain sensitivity associated with neurodegeneration, and these findings support a broader multi-regional view of early AD-associated transcriptional change (28). We performed behavioral pain assessments (von Frey and Hotplate tests) and found mechanical hypersensitivity and thermal hyperalgesia in 4–5-month-old APP/PS1 mice. However, detailed mechanistic studies and longitudinal phenotyping are beyond the scope of this transcriptomic study. Nonetheless, our multi-regional transcriptomic data highlight candidate molecular pathways and gene alterations that may underlie the observed nociceptive alterations and guide future diagnostic and therapeutic work aimed at preserving sensory function and slowing AD progression.

## 2 Materials and methods

### 2.1 Animals

Transgenic amyloid precursor protein (APP)/PS1 mice MMRRC Strain #034832-JAX; RRID: MMRRC_034832-JAX) modeling Alzheimer’s disease (AD) and wild-type (WT) C57BL/6 mice (Strain #:000664; RRID: IMSR_JAX:000664), both obtained from the Jackson Laboratory (Bar Harbor, ME, USA) and subsequently bred and maintained at the Rutgers University Animal Research Center until they reached 2 months old. For the experiments, we used an equal number of male and female APP/PS1 mice and their wild-type littermates, with both sexes represented in each experimental group. Mice were housed under standard conditions with controlled temperature, a 12-hour light/dark cycle, and ad libitum access to food and water. All animal procedures were conducted in compliance with institutional and national guidelines and approved by the Rutgers University Institutional Animal Care and Use Committee (IACUC). Efforts were made to minimize animal numbers and suffering.

### 2.2 Behavioral tests

Established behavioral assays were utilized to comprehensively evaluate cognitive functions and mechanical and heat pain sensitivity in mice, including the Novel Object Recognition (NOR) test, Y-maze test, Von Frey filament test, Hotplate test (29–35). For the NOR test, mice were exposed to two identical objects for 20 min in an arena during the training session on day one. Twenty-four hours after the training session, the mouse was put back in the arena for 5 min, with only one novel object replaced by a familiar one. The time spent exploring each object was recorded, and the discrimination index was calculated using EthoVision XT17 software (Noldus Information Technology, Virginia, USA). The discrimination index (DI) was calculated as the time spent exploring the novel object minus the time spent exploring the familiar object, divided by the total time spent exploring both objects. In the Y-maze test, each mouse was allowed to freely explore the three arms of the Y-maze for 5 minutes. The spontaneous alternation percentage in this Y-maze was analyzed using EthoVision XT17 software and calculated as the number of consecutive entries into all three different arms divided by the number of possible alternations (total arm entries minus 2). Mechanical sensitivity was assessed using calibrated von Frey filaments (0.07g and 0.4 g; Stoelting Co., Wood Dale, IL). Mice were placed in Plexiglas chambers on an elevated mesh screen, and each filament was applied 10 times to the plantar surface of the hind paw. The percentage paw withdrawal frequency was calculated as [number of paw withdrawals/10 trials] × 100. For the Hotplate test, mice were put in Plexiglas chambers on glass plates, and a radiant heat beam from a Model 336 Analgesia Meter (IITC Inc. Life Science Instruments, Woodland Hills, CA) was applied to the middle of the plantar surface of each hind paw. Latency to paw withdrawal was recorded, with a cutoff of 20 seconds to avoid tissue damage. All behavioral apparatuses were cleaned with 70% ethanol between trials to remove odor cues. The experimenter was blinded to the mouse strain and unaware of any expected behavioral differences. After behavioral tests, the DRG, spinal cord, thalamus, and hippocampus were rapidly collected under aseptic conditions. Tissues were immediately snap frozen in liquid nitrogen and stored at -80 °C until further analysis.

### 2.3 Whole transcriptome sequencing

Three biological replicates were prepared for each genotype (APP/PS1 and WT), resulting in a total of 24 samples for the subsequent whole transcriptome sequencing assay (4 regions × 2 genotypes × 3 replicates). Given that the tissues from one mouse were not sufficient for library preparation and RNA-seq assay, each RNA-seq biological replicate was generated by pooling tissue from 2 or 3 individual mice. These pooled samples were from age- and sex-matched mice of the same genotype. All samples were processed using the same library preparation protocol and sequenced under identical experimental conditions to minimize technical variability. High-quality total RNA was isolated from the hippocampus, spinal cord, dorsal root ganglion, and thalamus from both APP/PS1 and WT mice. RNA integrity and purity were evaluated using 1% agarose gel electrophoresis, the Agilent 2100 Bioanalyzer, and a NanoDrop spectrophotometer. DNase I treatment was performed to eliminate residual genomic DNA. For transcriptome sequencing, 1.5 μg of high-quality RNA per sample was used for library construction. Libraries were prepared with the NEBNext^®^ Ultra™ RNA Library Prep Kit for Illumina^®^ (NEB, USA) according to the manufacturer’s instructions. Briefly, RNA was enriched using poly-T oligo-attached magnetic beads, fragmented, and reverse transcribed to synthesize cDNA. After end repair, adaptor ligation, and size selection, PCR amplification was performed. Library quality was evaluated using the Agilent 2100 Bioanalyzer. The Illumina Novaseq 6000 platform was used for the sequencing, generating paired-end 150 bp reads.

### 2.4 Differential gene expression analysis

Differential gene expression analysis was performed with R package DESeq2 (1.48.1) in R version 4.5.0 to identify whether the genes were upregulated or downregulated between the APP/PS1 and WT mice. P-values were calculated using DESeq2, and the Benjamini-Hochberg correction method was applied to obtain adjusted P-values and control the false discovery rate. Volcano plots and bar graphs were generated using ggplot2 in R to visualize differentially expressed genes from RNA-seq datasets.

### 2.5 GO and KEGG enrichment analyses

Gene Ontology (GO) analysis was performed to identify the biological functions and processes in which these genes are involved. GO analysis includes three main categories: Biological Process (BP), Molecular Function (MF), and Cellular Component (CC). The GO enrichment analysis was performed using both the GOseq and clusterProfiler R packages in R version 4.5.0 (36), with annotation provided by the org.Mm.eg.db database. GOseq uses a statistical method known as the Wallenius noncentral hypergeometric distribution (37), which corrects for gene length bias: longer genes are more likely to be detected as differentially expressed, thereby ensuring greater accuracy. For GO enrichment analysis, we applied P-value and q-value cutoffs of 0.05 to define significant enrichment. The Kyoto Encyclopedia of Genes and Genomes (KEGG) is a biological database that uses large-scale datasets to help researchers understand how genes function within specific biological pathways (38). KEGG pathway enrichment analysis was performed using the KOBAS and clusterProfiler R packages to statistically evaluate and compare the differentially expressed genes to known KEGG pathways (39), allowing determination of which pathways were significantly associated with the differentially expressed genes. For KEGG pathway enrichment, we set the significance thresholds at P-value < 0.05 and q-value < 0.05.

### 2.6 Quantitative real-time RT-PCR (qRT-PCR) assay

To confirm the RNA-seq finding, we performed a quantitative real-time RT-PCR (qRT-PCR) assay. Primers were designed and obtained from Integrated DNA Technologies (IDT). The RNeasy Mini Kit (Qiagen, Cat. No. 74104) was used to isolate total RNA from the DRG, spinal cord, thalamus, and hippocampus of 4–5 months WT and APP/PS1 mice. The extracted RNA was reverse-transcribed into cDNA using the iScript cDNA Synthesis Kit (Bio-Rad, Cat. No. 1708890) according to the protocol provided. Quantitative real-time RT-PCR was performed using iTaq Universal SYBR Green Supermix (Bio-Rad) on a Bio-Rad CFX Opus 96 Real-Time PCR System. Each reaction was run in triplicate. Relative gene expression levels were calculated using the 2^-ΔΔCt method, with GAPDH as the endogenous control. CFX Maestro Software (Bio-Rad) was used for data acquisition and analysis. Statistical analysis was performed using GraphPad Prism, and data are presented as mean ± SEM from three independent experiments. Statistical significance between groups was determined using an unpaired Student’s t-test; P < 0.05 was considered significant. The nucleotide sequences of the primers used for qRT-PCR are listed in **Table 1**.

**Table 1:** Primer sequences used for qRT-PCR.

Primer	Direction	Sequence (5′ -> 3′)
GAPDH	Forward	AGGTCGGTGTGAACGGATTTG
	Reverse	TGTAGACCATGTAGTTGAGGTCA
Plin4	Forward	GTTTCTTTGGGTCCCTGCCT
	Reverse	TTCAGAAGGTTGGAGCAGCC
Rps3a3	Forward	ATGCAGTTGTAGCTTTTGGCT
	Reverse	TGGTTCCTTGAGTCCTCGTG
Hmga1b	Forward	CTCCGAGTCGGGGCTATTTC
	Reverse	CTCGCTTCTCAGTCCCATCC
Prnp	Forward	TCGGATCAGCAGACCGATTC
	Reverse	TGACTGATAGTCTGCTTGGCT

### 2.7 Detection of β-amyloid plaques

Hemibrain sections (30 μm thickness, six brains per group) were incubated twice in 70% ethanol for 1 minute each to prepare the tissue for β-amyloid plaque detection. Sections were then stained with a 0.5% Thioflavin S solution prepared in 50% ethanol (ethanol:ddH2O, 1:1) for 15 minutes. To remove nonspecifically bound dye and retain amyloid fluorescence, the sections were rinsed twice in 70% ethanol for 1 minute each, followed by 2 rinses with distilled water. Aqueous mounting medium was used to mount the sections. The sections were imaged using a fluorescence microscope (Olympus BX53M). Thioflavin S-positive Aβ plaques were quantified in the hippocampus and cortex using Fiji. For each brain section, the regions of interest (ROIs) corresponding to the hippocampus and cortex were delineated, and the total ROI area was measured in mm^2^. Aβ plaques were identified using a consistent fluorescence threshold across experimental groups. The number of Aβ plaques within each ROI was counted, and plaque density was calculated as: Plaque density (plaques/mm^2^) = Number of Aβ plaques/ROI area (mm^2^).

### 2.8 Measurement of TNF-α and IL-1β levels

TNF-α and IL-1β levels were assessed in the DRG, spinal cord, thalamus, and hippocampus tissues from 4- to 5-month-old wild-type (WT) and APP/PS1 mice to evaluate inflammation. Tissue lysates were prepared and analyzed according to the manufacturer’s protocol. TNF-α and IL-1β concentrations were quantified using the Tribo™ Mouse TNF-α ELISA Kit (TribioScience Inc., Cat. No. TBS3030) and the Tribo™ Mouse IL-1β ELISA Kit (TribioScience Inc., Cat. No. TBS3050). Briefly, the samples, standards, and controls were incubated in ELISA plate wells with detection reagents for 2 hours at room temperature, followed by a 1-hour incubation with secondary detection reagent after being washed. TMB substrate was then added and incubated for 20 minutes at room temperature in the dark before the reaction was stopped. Absorbance was measured at 450 nm using a microplate reader. All measurements were performed in triplicate.

### 2.9 Immunoblot assay

Tissues were lysed in RIPA buffer containing 1 mM PMSF and sonicated on ice. Lysates were centrifuged at 13,000 × g for 12 minutes at 4 °C, and supernatants were collected for protein quantification using the Pierce™ BCA Protein Assay Kit (Thermo Fisher Scientific, Cat. No. A55865). Equal amounts of protein (10 μg per sample) were separated by 10% SDS-PAGE and transferred to 0.45 μm PVDF membranes. Membranes were blocked with 3% BSA in TBST for 1 hour at room temperature, then incubated with primary antibodies, anti-PSD95 (Abcam, ab18258; 1:1000), anti-Synaptophysin (Abcam, Cat. No. ab8049; 1:7500), and anti-β-actin (Thermo Fisher Scientific, Cat. No. MA1-140; 1:5000), for 2 hours at room temperature. After three washes with TBST, membranes were incubated with HRP-conjugated secondary antibodies, rabbit anti-Mouse IgG (H+L) (Thermo Fisher Scientific, Cat. No. 31450) and goat anti-Rabbit IgG (H+L) (Thermo Fisher Scientific, Cat. No. 31460), for 1 hour at room temperature, followed by three additional washes with TBST. Protein bands were visualized using enhanced chemiluminescence (ECL) reagent (Thermo Fisher Scientific, Cat. No. 32106), and images were acquired with a Bio-Rad ChemiDoc MP Imaging System. Band intensities were quantified using ImageJ software and normalized to beta-actin.

### 2.10 Statistical analysis

All experiments were performed at least three times independently. Data are shown as mean ± SEM, and the sample size (n) is specified for each experiment. Analyses were performed with GraphPad Prism 10 and R v4.5.0. Normality of data distribution was assessed prior to analysis. For comparisons between two independent groups, the unpaired Student’s t-test was used when the assumption of normality was satisfied. All statistical analyses were set with a significance threshold of 0.05.

## 3 Results

### 3.1 Cognitive behaviors in the early stage of APP/PS1 Mice

**Figure 1:**
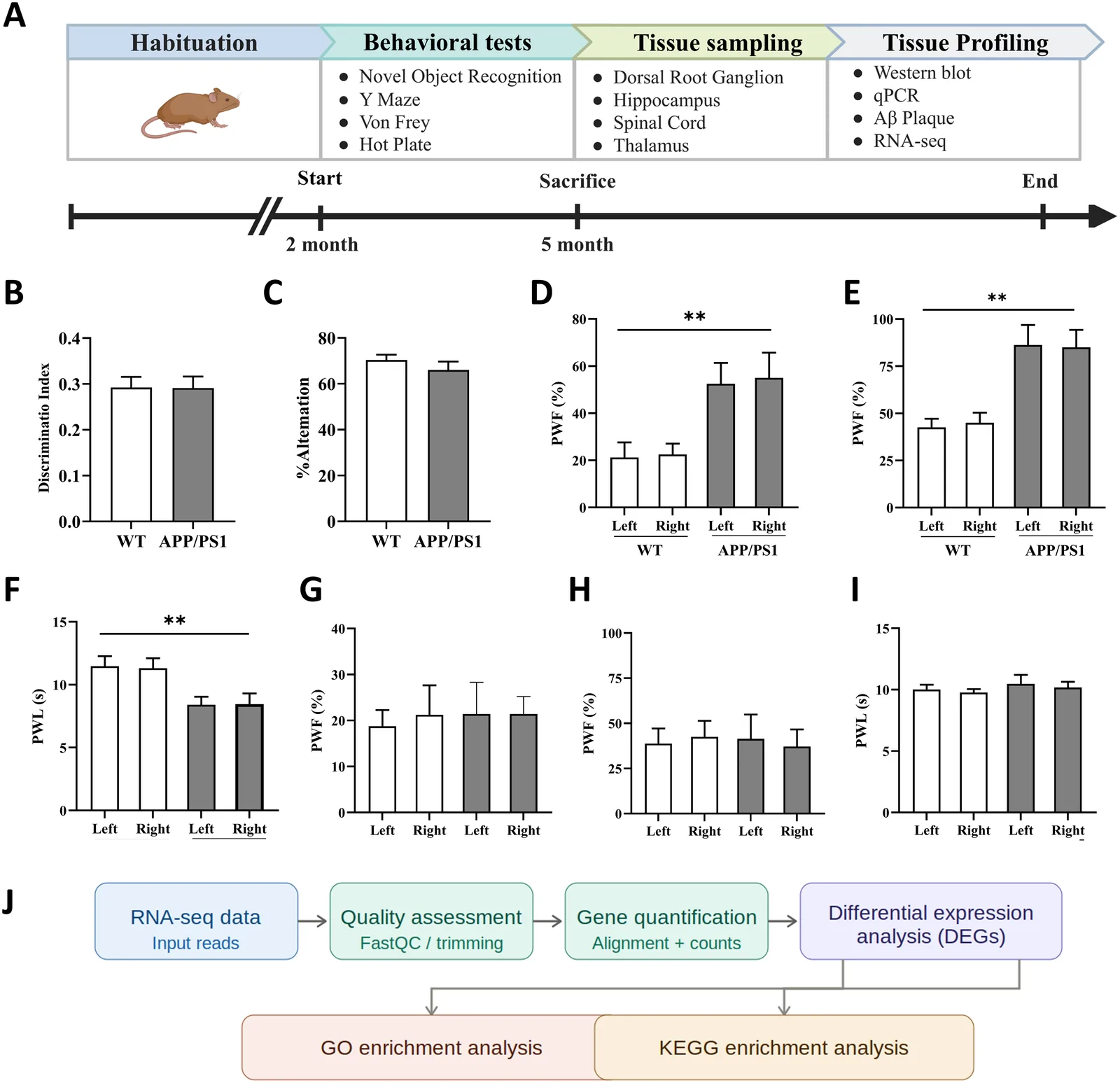
Behavioral and experimental timeline with behavioral assessment. **(A)** Schematic paradigm of experimental design, showing habituation, behavioral assessments, tissue sampling, and molecular profiling. **(B–F)** Behavioral results from 4–5-month-old mice: **(B)** Novel object recognition (NOR) test. **(C)** Y-maze test. **(D, E)** Mechanical sensitivity assessed using Von Frey filaments with bending forces of 0.07 g and 0.4 g, respectively; **(F)** Thermal nociception measured by Hotplate test (paw-withdrawal latency, s). **(G–I)** Behavioral results from 2-month-old mice: **(G)** Von Frey 0.07 g; **(H)** Von Frey 0.4 g; **(I)** Hotplate test. **(J)** Analysis pipeline for RNA sequencing data. Data are shown as mean ± SEM (n = 7–8 per group for behavioral tests).

As outlined in the experimental timeline (**Figure 1A**), a series of behavioral tests was conducted in 4–5 months APP/PS1 mice to evaluate early cognitive and nociceptive changes. It is well documented that APP/PS1 mice begin to exhibit changes in cognitive and memory behaviors starting from seven months old (40). Prior to this stage, their memory and cognitive behaviors do not differ from those of their age-matched wild-type mice. As expected, the novel object recognition (NOR) test showed that the discrimination index (DI) of wild-type mice at 4–5 months was around 0.3 and that the APP/PS1 mice did not show significant changes in DI when compared to these wild-type control littermates, indicating that the APP/PS1 mice at this age did not show the detectable memory impairment at this age (**Figure 1B**). Similarly, the subsequent Y-maze test revealed that the percentage of spontaneous alteration in the APP/PS1 mice was about 60%, which did not show a significant difference when compared with age-matched wild-type controls (**Figure 1C**), suggesting that these APP/PS1 mice did not exhibit spatial memory loss at 4–5 months old. These results confirm that subsequent molecular analyses were conducted during the early pathological stage, allowing us to identify early transcriptomic changes in sensory and memory-related neural regions that preceded detectable cognitive dysfunction.

In contrast, nociceptive sensitivity was altered in 4–5-month-old APP/PS1 mice. The von Frey filament test is a classical approach used to detect static mechanical sensitivity, whereas the hotplate test is employed to measure heat sensitivity (41). The APP/PS1 mice at 4–5 months of age showed significantly higher paw withdrawal frequencies in the responses to 0.07 and 0.4 g von Frey filament stimuli as compared to those age-matched WT (**Figures 1D, E**). The paw withdrawal latencies to heat stimuli in the APP/PS1 mice were significantly lower than those of age-matched WT littermates (**Figure 1F**). To establish whether these sensory alterations were present earlier, we tested a separate 2-month-old cohort. The results showed that 2-month-old APP/PS1 mice did not differ from age-matched WT controls in paw-withdrawal frequency to either 0.07 g or 0.4 g von Frey filaments (**Figures 1G, H**), nor in paw-withdrawal latency on the hotplate test (**Figure 1I**). No left/right side differences were observed during the experiments. These comparisons were not significant (p > 0.05; two-way ANOVA; n = 7–8 per group), indicating that the mechanical hypersensitivity and thermal hyperalgesia observed at 4–5 months are not present at 2 months of age. These results indicate that cognitive function remains intact at 4–5 months, while nociceptive threshold alterations develop during the early stage of the APP/PS1 model. These findings indicate that nociceptive hypersensitivity emerges before detectable cognitive impairment in 4–5-month-old APP/PS1 mice, providing a behavioral basis for investigating early molecular changes in pain-related tissues.

### 3.2 Quality assessment and principal component analysis

RNA-Seq samples from the DRG, spinal cord, thalamus, and hippocampus of both APP/PS1 mice and age-matched WT littermates underwent thorough quality control to ensure the reliability and repeatability of our transcriptomic results. For each group, biological replicates were included to account for inherent biological variability and enhance the reliability of downstream analyses. **Figure 1J** shows the overall selection strategy and analytical workflow, while the following subsections provided comprehensive descriptions of each analytical stage.

**Figure 2:**
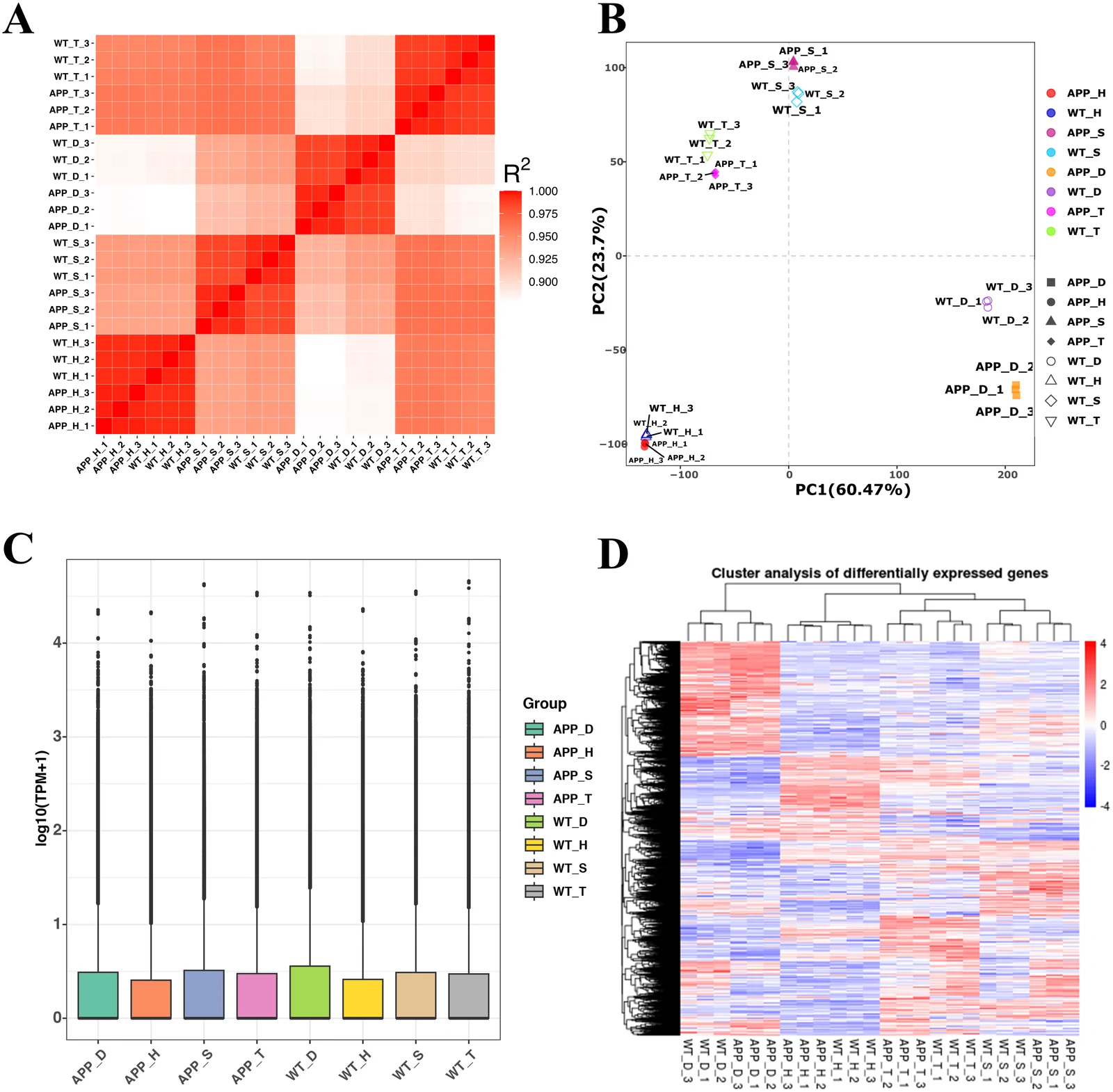
Quality assessment of RNA sequencing data. **(A)** Heatmap showing the Spearman correlation coefficients (R²) for all samples; the numbers in each cell indicate the square of the correlation coefficient between the corresponding samples. Higher R² (darker red) indicates greater similarity. **(B)** Principal component analysis (PCA) plot of all samples. **(C)** Boxplot showing the distribution of log10(TPM + 1) values for each sample group. **(D)** Hierarchical clustering heatmap of differentially expressed genes (DEGs) across all samples. Sample names indicate genotype and tissue type: WT and APP represent wild-type and APP/PS1 transgenic samples, respectively; D, H, S, and T indicate dorsal root ganglion, hippocampus, spinal cord, and thalamus, respectively; and the numeric suffix (e.g., _1, _2, _3) denotes biological replicates.

To assess data quality, we used pairwise Spearman’s correlation analysis to examine gene expression profiles across biological replicates for each tissue and experimental condition. Higher R² values indicated greater similarity among samples. In our study, intra-group correlations were consistently high, with R² values above 0.8, indicating strong concordance among biological replicates and supporting the overall consistency of the RNA-seq data (**Figure 2A**). Additionally, principal component analysis (PCA) was performed to further investigate the variance structure and sample relationships within the transcriptomic dataset. By efficiently reducing high-dimensional gene expression data to principal components, PCA enables visualization of sample clustering and identification of outliers. When we examined the sample distribution along the first pair of primary groups, we discovered that biological replicates within the same tissue and experimental group clustered closely together. On the other hand, samples from different tissues showed clear differentiation. These results were consistent with overall good-quality RNA-seq data and supported the suitability of the samples for downstream differential expression analyses (**Figure 2B**). To evaluate overall expression patterns, raw counts were imported into R and converted to TPM (Transcripts Per Million) in the R programming language. TPM values were computed for each gene across all samples to facilitate cross-sample expression comparisons, including DRG, spinal cord, thalamus, and hippocampus tissues from APP/PS1 and WT mice. For each gene in each sample TPM = (RPK/sum (RPK)) × 10^6, where RPK (Reads Per Kilobase) = counts/(length_kb). Box plots were used to provide a thorough comparison of the expression distributions across all groups. Per-sample and per-group boxplots of log10(TPM + 1) were produced with the ggplot2 package in R. After computing TPM (and excluding genes lacking length), per-sample and per-group boxplots of log10(TPM + 1) show broadly similar global expression distributions across experimental clusters, indicating no major library-size or composition bias between groups. The results showed similar general patterns of gene expression across all tissue types and genotypes, which further validated the quality and comparability of the data (**Figure 2C**). Differentially expressed genes were categorized using hierarchical clustering analysis based on common expression patterns across experimental groups (**Figure 2D**). Representative raw-read QC metrics for full sequencing statistics of all samples are provided in **Supplementary Table 1**. This study further validated the biological importance of transcriptomic data by identifying distinct gene expression profiles associated with genotypes and regions.

### 3.3 Identification of differentially expressed genes (DEGs)

**Figure 3:**
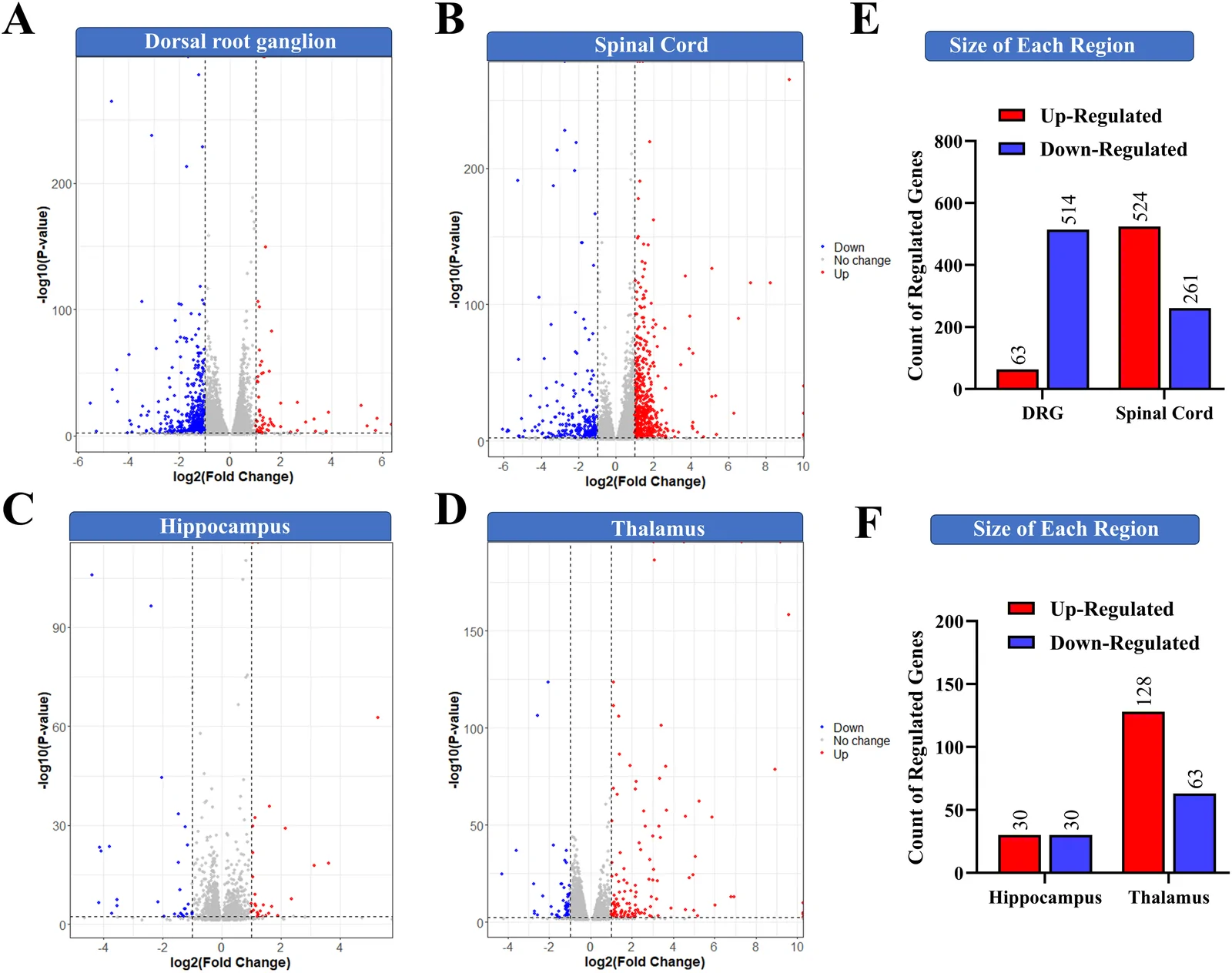
Volcano plots and summary of differentially expressed genes in each region. **(A)** Dorsal root ganglion, **(B)** spinal cord, **(C)** hippocampus, and **(D)** thalamus. Red dots indicate upregulated genes, blue dots indicate downregulated genes, and gray dots indicate genes with no significant change. The X-axis represents log_2_(fold change), and the Y-axis represents –log_10_(P-value). **(E, F)** Bar graphs show the number of upregulated (red) and downregulated (blue) DEGs in each region.

To elucidate region-specific transcriptional alterations associated with AD pathology, we conducted transcriptomic profiling of the DRG, spinal cord, thalamus, and hippocampus in the APP/PS1 and WT mice. By applying criteria (|log2 fold change| > 1, Benjamini-Hochberg adjusted P-value < 0.005) to prioritize robust region-shared candidates across tissues, DEGs were identified in all regions, and pseudogenes were not included in the analysis. For every tissue, volcano plots showed the distribution and scope of changes in gene expression (**Figures 3A–D**). In the dorsal root ganglion (**Figure 3A**), we identified 63 upregulated and 514 downregulated genes. The spinal cord (**Figure 3B**) showed 524 upregulated and 261 downregulated genes. While the hippocampus (**Figure 3C**) exhibited 30 upregulated and 30 downregulated genes, the thalamus (**Figure 3D**) showed 128 upregulated and 63 downregulated genes. In these plots, red dots represent upregulated genes, blue dots represent downregulated genes, and gray dots indicate genes without significant change. Bar graphs (**Figures 3E, F**) summarizing the overall numbers of up- and down-regulated differentially expressed genes (DEGs) across regions further highlighted notable region-specific variations, especially the transcriptional dysregulation observed in the spinal cord and dorsal root ganglia of APP/PS1 mice. Notably, the spinal cord and DRG showed the largest transcriptional changes, suggesting a stronger peripheral and spinal response at this early stage. Together, these findings showed apparent region-specific alterations in gene expression in APP/PS1 mice, indicating distinct molecular effects of AD development across diverse regions. **Supplementary Table 2** (DRGs, hippocampus, spinal cord, thalamus) provides complete post-treatment DEG lists (|log2FC| > 1, BH-adjusted P < 0.005; pseudogenes removed) comparing APP/PS1 with WT.

### 3.4 GO and KEGG enrichment analyses

We conducted KEGG pathway and GO enrichment analyses on differentially expressed genes across the dorsal root ganglia, spinal cord, hippocampus, and thalamus to determine the functional significance of transcriptional changes in APP/PS1 mice. All these analyses showed that DEGs in these tissue regions from the APP/PS1 mice were primarily involved in immune response, ion transport, neural signaling, synaptic function, and membrane organization. The results revealed both region-specific and shared molecular mechanisms underlying AD pathology. **Supplementary Table 3** shows GO/KEGG enrichments of DEGs in the dorsal root ganglion, hippocampus, spinal cord, and thalamus for the APP/PS1 group compared to WT.

**Figure 4:**
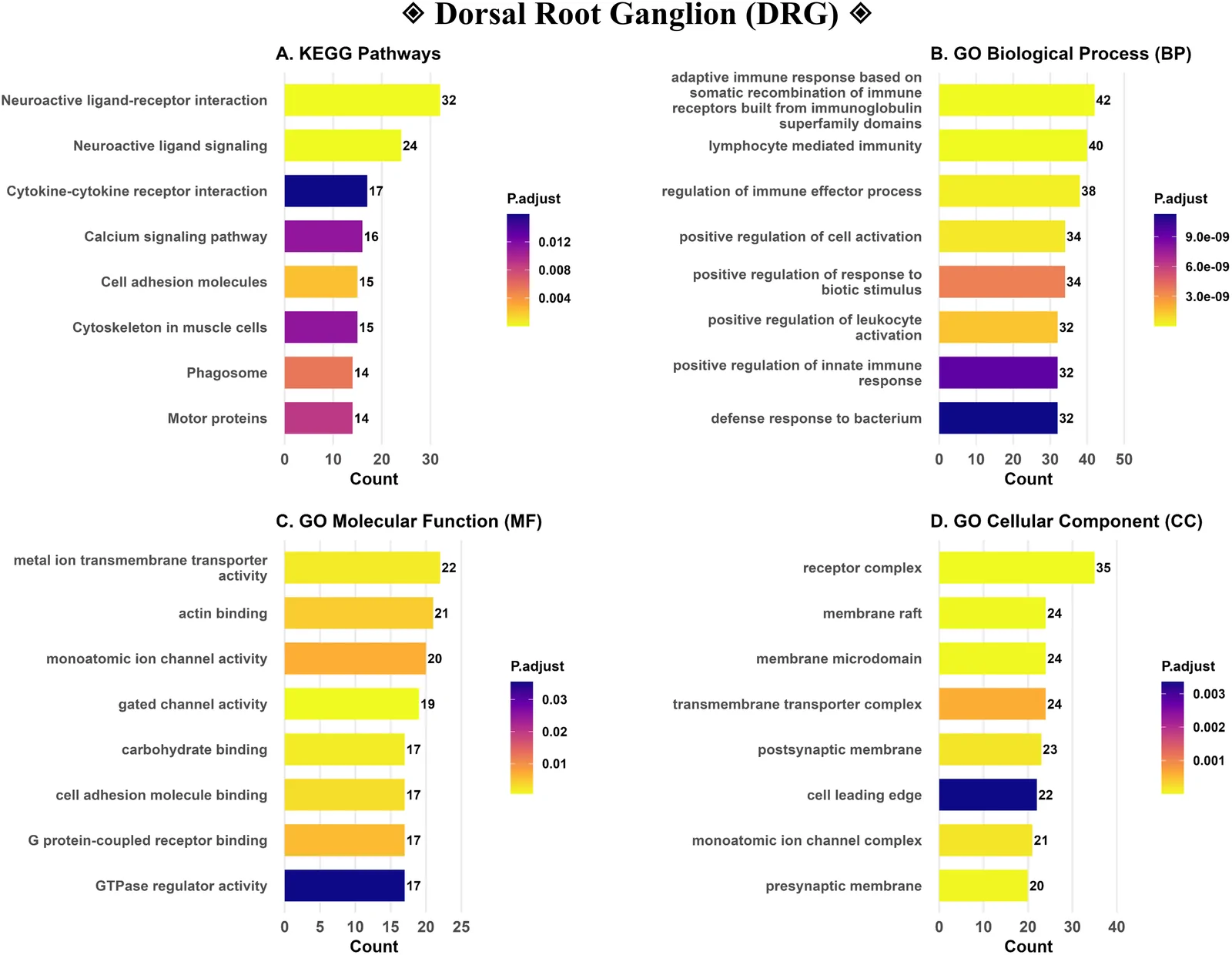
KEGG pathway and GO enrichment analysis of differentially expressed genes in the dorsal root ganglion. **(A)** Top 8 enriched KEGG pathways. **(B)** GO biological processes (BP). **(C)** GO molecular functions (MF). **(D)** GO cellular components (CC) for DEGs in the dorsal root ganglion. The bar length indicates gene count, and bar color indicates adjusted P-value.

In the DRG, a total of 577 DEGs (63 upregulated and 514 downregulated) were analyzed. KEGG pathway analysis (**Figure 4A**) indicated that the DEGs were mainly enriched in neuroactive ligand-receptor interaction, neuroactive ligand signaling, cytokine-cytokine receptor interaction, calcium signaling, cell adhesion molecules, phagosome, and motor protein-related pathways. GO biological process (BP) analysis (**Figure 4B**) showed prominent enrichment in immune-related processes, including adaptive immune response, lymphocyte-mediated immunity, regulation of immune effector process, positive regulation of cell activation, positive regulation of leukocyte activation, and defense response to bacterium. GO molecular function (MF) analysis (**Figure 4C**) highlighted metal ion transmembrane transporter activity, actin binding, monoatomic ion channel activity, gated channel activity, cell adhesion molecule binding, and G protein-coupled receptor binding. GO cellular component (CC) analysis (**Figure 4D**) showed enrichment in receptor complex, membrane raft, membrane microdomain, transmembrane transporter complex, postsynaptic membrane, monoatomic ion channel complex, and presynaptic membrane. Together, these results suggest that APP/PS1 pathology in the DRG is associated with disrupted immune signaling and altered membrane excitability, which may contribute to abnormal peripheral nociceptive processing.

**Figure 5:**
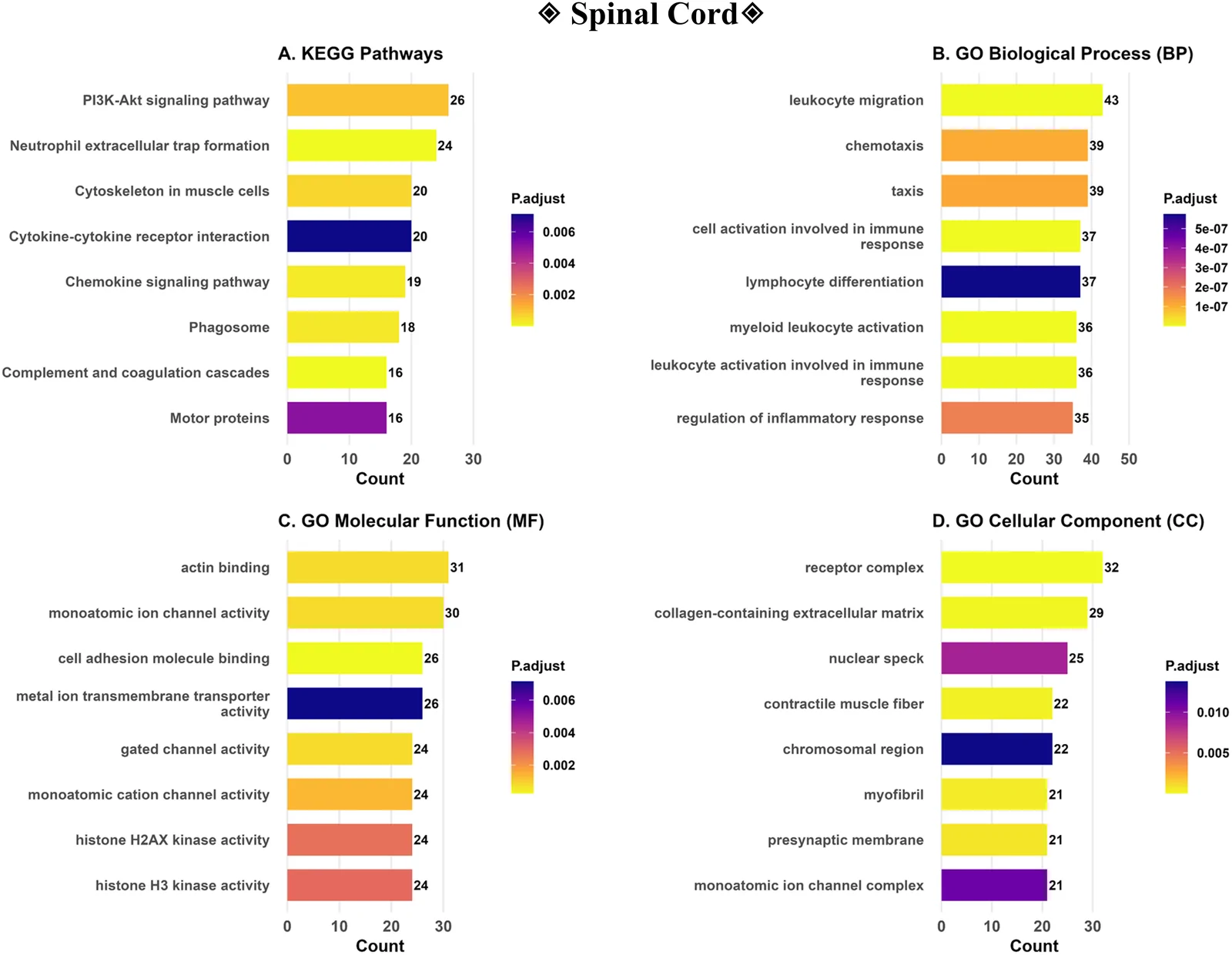
KEGG pathway and GO enrichment analysis of differentially expressed genes in the spinal cord. **(A)** Top 8 enriched KEGG pathways. **(B)** GO biological processes (BP). **(C)** GO molecular functions (MF). **(D)** GO cellular components (CC) for DEGs in the spinal cord. The bar length indicates gene count, and bar color indicates adjusted P-value.

In the spinal cord, 785 DEGs (524 upregulated and 261 downregulated) were analyzed. KEGG pathway analysis (**Figure 5A**) revealed enrichment in PI3K-Akt signaling, neutrophil extracellular trap formation, cytokine-cytokine receptor interaction, chemokine signaling, phagosome, complement and coagulation cascades, and motor protein-related pathways. GO BP analysis (**Figure 5B**) showed that the DEGs were mainly associated with leukocyte migration, chemotaxis, taxis, cell activation involved in immune response, lymphocyte differentiation, myeloid leukocyte activation, leukocyte activation involved in immune response, and regulation of inflammatory response. GO MF analysis (**Figure 5C**) indicated enrichment in actin binding, monoatomic ion channel activity, cell adhesion molecule binding, metal ion transmembrane transporter activity, gated channel activity, and histone kinase activity. GO CC analysis (**Figure 5D**) showed enrichment in receptor complex, collagen-containing extracellular matrix, nuclear speck, contractile muscle fiber, chromosomal region, myofibril, presynaptic membrane, and monoatomic ion channel complex. These findings indicate that the spinal cord transcriptome is strongly shaped by immune activation and inflammatory signaling, together with changes in ion-channel and synaptic-related functions, which may contribute to altered nociceptive transmission.

**Figure 6:**
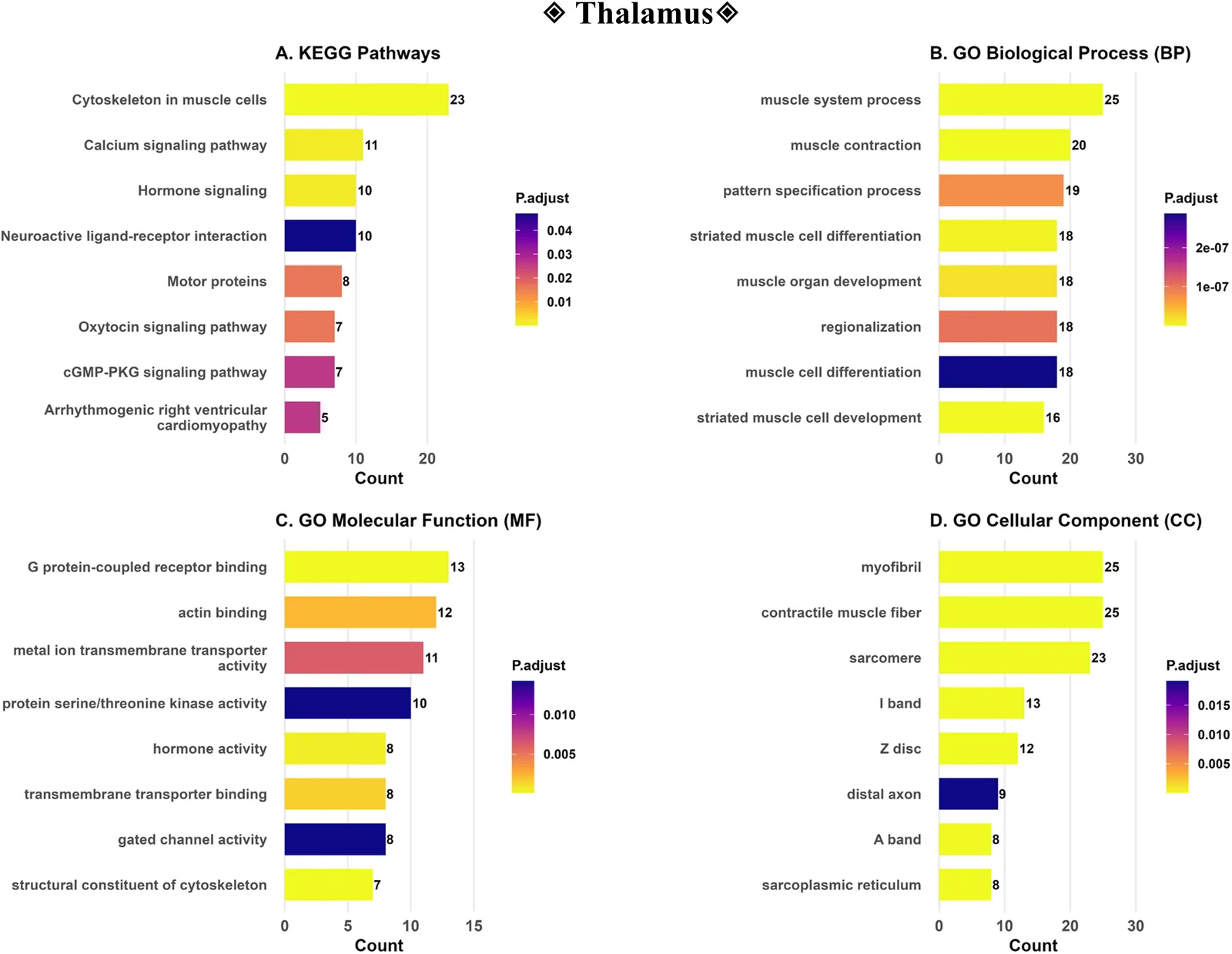
KEGG pathway and GO enrichment analysis of differentially expressed genes in the thalamus. **(A)** Top 8 enriched KEGG pathways. **(B)** GO biological processes (BP). **(C)** GO molecular functions (MF). **(D)** GO cellular components (CC) for DEGs in the thalamus. The bar length indicates gene count, and bar color indicates adjusted P-value.

In the thalamus, 191 DEGs (128 upregulated and 63 downregulated) were analyzed. KEGG pathway analysis (**Figure 6A**) revealed enrichment in the cytoskeleton in muscle cells, calcium signaling, hormone signaling, neuroactive ligand-receptor interaction, motor proteins, oxytocin signaling, cGMP-PKG signaling, and arrhythmogenic right ventricular cardiomyopathy. GO BP analysis (**Figure 6B**) showed enrichment in muscle system process, muscle contraction, pattern specification process, striated muscle cell differentiation, muscle organ development, regionalization, muscle cell differentiation, and striated muscle cell development. GO MF analysis (**Figure 6C**) indicated enrichment in G protein-coupled receptor binding, actin binding, metal ion transmembrane transporter activity, protein serine/threonine kinase activity, hormone activity, transmembrane transporter binding, gated channel activity, and structural constituent of cytoskeleton. GO CC analysis (**Figure 6D**) showed enrichment in myofibril, contractile muscle fiber, sarcomere, I band, Z disc, distal axon, A band, and sarcoplasmic reticulum. Overall, this enrichment patterns suggest altered signaling and cytoskeletal organization in the thalamus.

**Figure 7:**
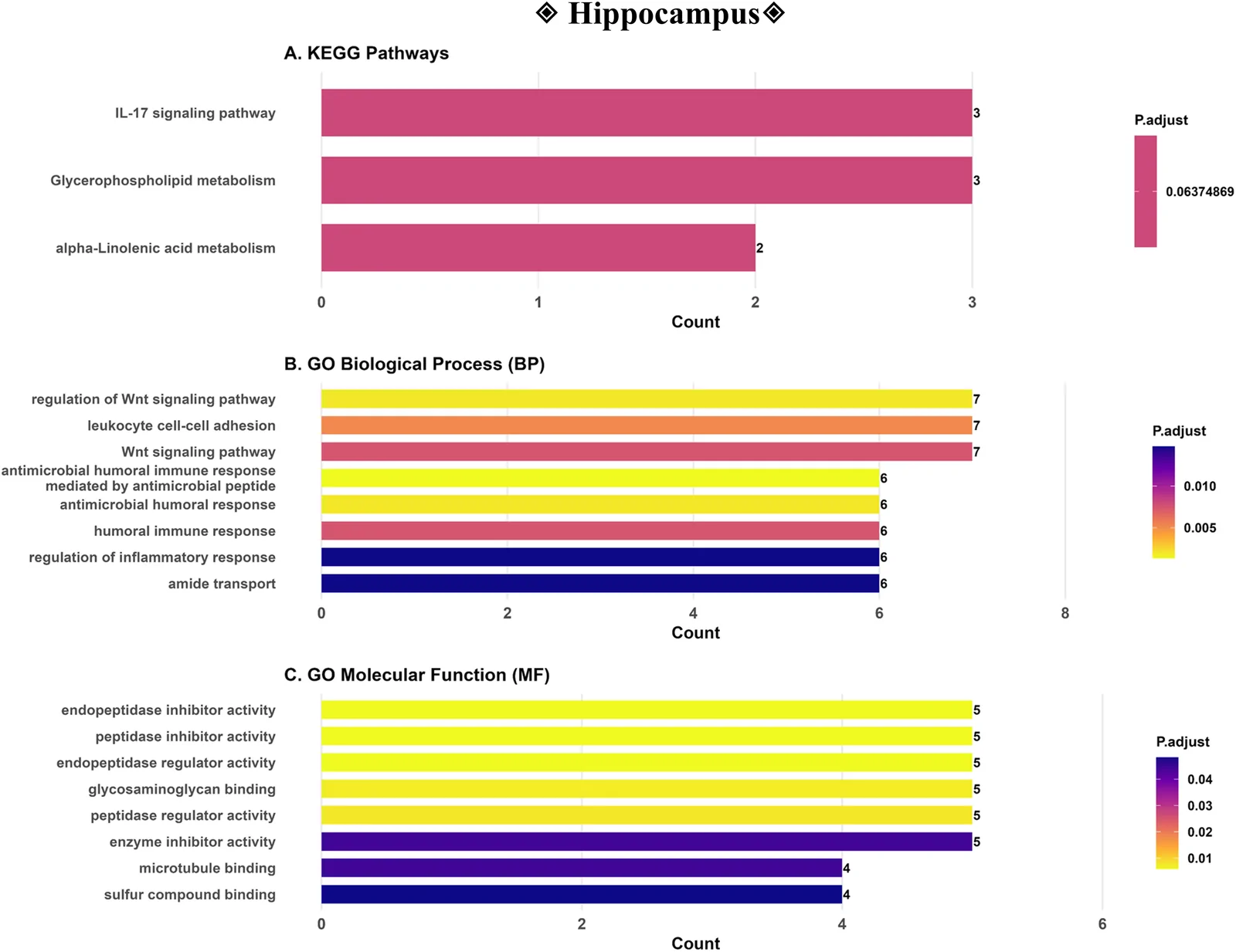
KEGG pathway and GO enrichment analysis of differentially expressed genes in the hippocampus. **(A)** Top 8 enriched KEGG pathways. **(B)** GO biological processes (BP). **(C)** GO molecular functions (MF) for DEGs in the hippocampus. The bar length indicates gene count, and bar color indicates adjusted P-value.

In the hippocampus, 60 DEGs (30 upregulated and 30 downregulated) were identified. KEGG pathway analysis (**Figure 7A**) showed enrichment in IL-17 signaling, glycerophospholipid metabolism, and alpha-linolenic acid metabolism. GO BP analysis (**Figure 7B**) indicated enrichment in Wnt signaling-related processes, leukocyte cell-cell adhesion, humoral immune response, regulation of inflammatory response, and amide transport. GO MF analysis (**Figure 7C**) revealed enrichment in endopeptidase inhibitor activity, peptidase inhibitor activity, peptidase regulator activity, glycosaminoglycan binding, enzyme inhibitor activity, microtubule binding, and sulfur compound binding. Overall, these results suggest that hippocampal transcriptional changes in APP/PS1 mice involve inflammatory signaling together with pathways related to membrane metabolism and structural regulation.

Overall, the enrichment analyses revealed tissue-specific transcriptional signatures in APP/PS1 mice. In the DRG and spinal cord, the DEGs were predominantly enriched in immune-related pathways, cytokine signaling, leukocyte activation or migration, and receptor/ion channel functions. In the hippocampus, enrichment was observed in inflammatory, Wnt-related, and lipid metabolism-associated processes, whereas the thalamus showed enrichment in signaling, muscle-related, and cytoskeletal terms. Together, these data indicate that early APP/PS1 pathology is accompanied by region-specific molecular remodeling in pain- and cognition-related tissues.

### 3.5 Overlap analysis of differentially expressed genes

**Figure 8:**
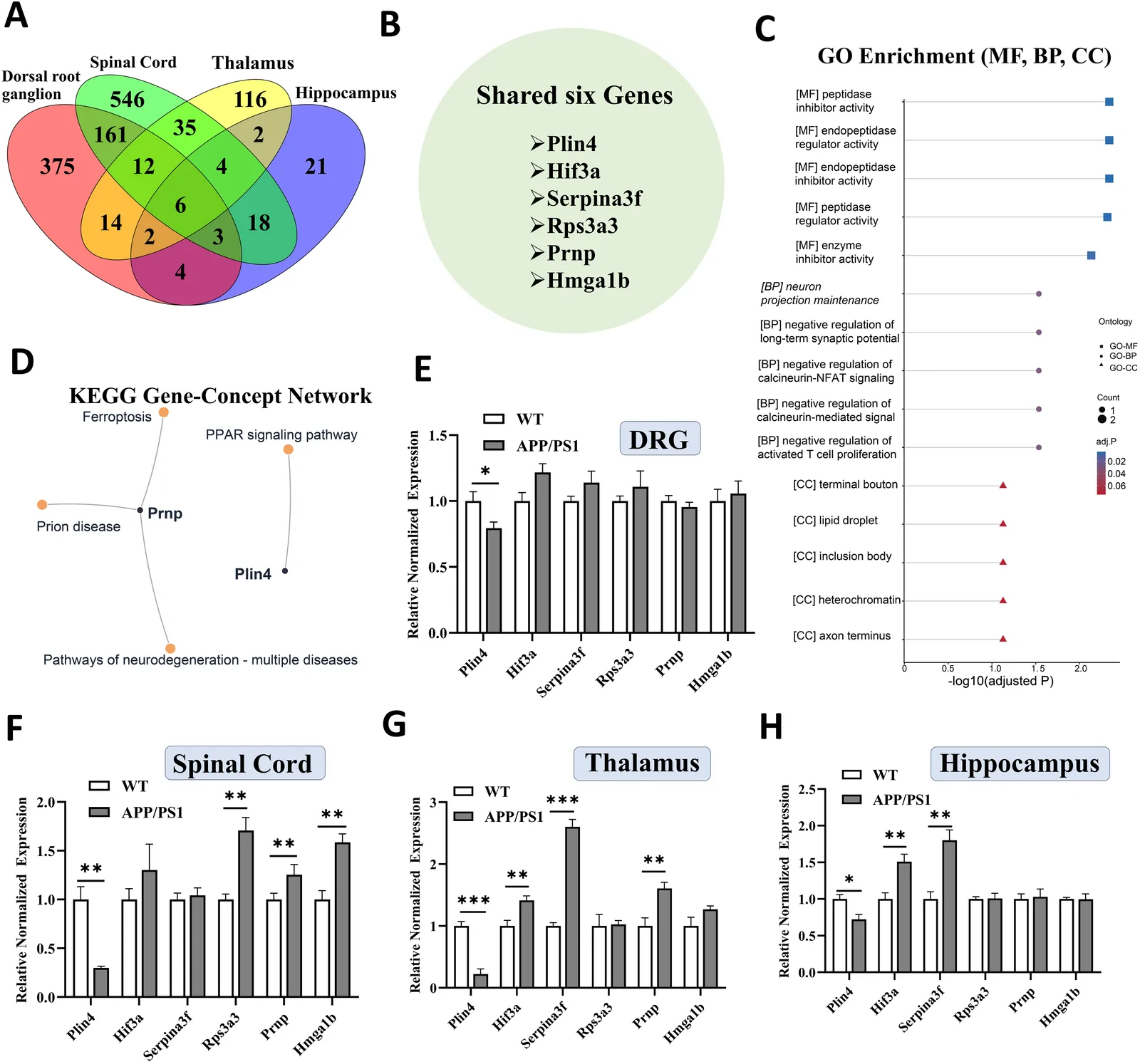
Overlap analysis of differentially expressed genes and quantitative real-time RT-PCR (qRT-PCR) validation. **(A)** Four-way Venn diagram showing the number of DEGs unique to and shared among DRG, spinal cord, thalamus, and hippocampus. **(B)** The intersection lists the genes commonly regulated in four regions. **(C)** Gene Ontology (GO) enrichment analysis (Molecular Function, Biological Process, Cellular Component) of the shared DEGs. **(D)** KEGG gene–concept network highlighting pathways. Analysis of *Plin4, Hif3a, Serpina3f, Rps3a3, Prnp, and Hmga1b* gene mRNA levels in the **(E)** DRG, **(F)** spinal cord, **(G)** thalamus, and **(H)** hippocampus of WT and APP/PS1 mice. Significant differences between WT and APP/PS1 groups are indicated (*P < 0.05, **P < 0.01; unpaired t-test). Relative normalized expression levels are shown as mean ± SEM. (N = 6).

To determine common transcriptional signatures, we compared differentially expressed genes among the hippocampus, thalamus, dorsal root ganglion, and spinal cord (**Figures 8A–D**). While there were some DEG overlaps among these four regions, Venn diagram comparisons showed that most DEGs were specific to each region. Remarkably, only six genes were consistently differentially expressed: Plin4, Hif3a, Rps3a3, Serpina3f, Hmga1b, and Prnp (**Figures 8A, B**). The overlapped DEGs across all regions emphasized how transcriptional changes in APP/PS1 mice were distinctly region-specific. These six genes are candidate early pathological signatures shared across pain and AD-related disease regions. The functional enrichment analysis of the six-gene set identified coherent biological themes across GO and KEGG. In the GO Biological Process, Prnp-associated neuronal and synaptic regulatory programs were enriched, including neuron projection maintenance and negative regulation of long-term synaptic potentiation (all adjusted P = 0.0292 among top BP terms). In GO Molecular Function, the strongest signal was inhibitory protease-related activity, with endopeptidase inhibitor activity as the top term (adjusted P = 0.00459), followed by peptidase inhibitor/regulator functions (adjusted P range: 0.00459-0.00736). These MF terms were primarily supported by Prnp and Serpina3f. KEGG analysis identified enrichment of two pathways, Ferroptosis (adjusted P = 0.0294), and the PPAR signaling pathway (adjusted P = 0.0318). In contrast, prion disease (adjusted P = 0.0645) and pathways of neurodegeneration - multiple diseases (adjusted P = 0.0844) showed suggestive but non-significant enrichment after multiple-testing correction. GO Cellular Component results were overall more moderate; lipid droplet (linked to Plin4) was among the top terms (adjusted P = 0.0747), indicating a trend-level signal for lipid compartment biology. Overall, these findings suggested that Prnp displayed broader enrichment across neuronal and inhibitory functional terms, whereas Plin4 showed a more focused association with lipid droplet and PPAR-related pathways. Together, these findings identify Prnp- and Plin4-associated pathways as potential areas for further investigation, particularly in relation to neuronal homeostasis and lipid metabolism. The results are summarized in **Supplementary Table 4**.

### 3.6 qRT-PCR validation of selected differentially expressed genes

To validate the transcriptomic findings, qRT-PCR was conducted to assess the expression levels of the genes (Plin4, Hif3a, Serpina3f, Rps3a3, Prnp, and Hmga1b) in the DRG, spinal cord, thalamus, and hippocampus of APP/PS1 and WT mice (**Figures 8E–H**). In the DRG, Plin4 expression was significantly decreased in APP/PS1 mice compared to WT (P < 0.05), while expression of the other genes remained unchanged (**Figure 8E**). In the spinal cord, Plin4 was again significantly downregulated in APP/PS1 mice (P < 0.05). In contrast, Rps3a3, Prnp, and Hmga1b were each significantly upregulated in APP/PS1 mice compared to WT in the spinal cord (P < 0.05, **Figure 8F**). In the Thalamus, Plin4 expression was significantly decreased in APP/PS1 mice compared to WT (P < 0.05), and Hif3a, Serpina3f, and Prnp were increased (**Figure 8G**). In the hippocampus, Plin4 was significantly downregulated in APP/PS1 mice relative to WT (P < 0.05). In contrast, Hif3a and Serpina3f were significantly increased in APP/PS1 mice relative to WT (**Figure 8H**). Overall, qRT-PCR confirmed the direction and significance of the RNA-seq result for Plin4 across DRG, spinal cord, thalamus, and hippocampus, and validated Rps3a3, Prnp, and Hmga1b increases specifically in the spinal cord. As well as the upregulation of Hif3a, Serpina3f, and Prnp in the thalamus and Hif3a and Serpina3f in the hippocampus. Several candidate genes exhibited directional trends consistent with the RNA-seq findings despite not reaching statistical significance in qRT-PCR assays. Notably, upward trends for Hif3a in the DRG and spinal cord, and for Serpina3f in the DRG, potentially reflect variability in effect size across samples.

### 3.7 Cross-species validation of candidate genes in human AD transcriptomic datasets

To assess the translational relevance of the six genes consistently dysregulated across the four mouse brain regions, we referenced a previous human AD transcriptomic study. We extracted our data directly from the **Supplementary File**, as it already provides the differential expression results (42). The human AD datasets included GSE33000, GSE48350, GSE5281, and GSE112063. We identified the corresponding human orthologs of the six mouse candidate genes as HIF3A, HMGA1, PLIN4, PRNP, RPS3A, and SERPINA3 and extracted the reported differential-expression results. The reported log2 fold change (log2FC) values and adjusted P values were compiled across datasets, and the mean, minimum, and maximum log2FC values were calculated for each human ortholog. Importantly, all six human orthologs exhibited statistically significant differential expression (adjusted P < 0.05) across the analyzed datasets. The direction of the reported human AD-associated expression change was then compared with that observed by mouse qRT-PCR. The complete dataset-level comparison is provided in **Supplementary Table 5**. Of the six candidate genes, four (HIF3A, HMGA1, RPS3A, and SERPINA3) showed directionally concordant expression changes between the reported human AD datasets and our mouse qRT-PCR results. HIF3A showed a mean human log2FC of +0.479 across three datasets (range: +0.295 to +0.830), while HMGA1 showed a mean log2FC of −0.222 across three datasets (range: −0.299 to −0.069). RPS3A showed a mean log2FC of +0.189 across two datasets (range: +0.029 to +0.349). Notably, SERPINA3 exhibited the strongest human AD-associated increase, with a mean log2FC of +1.577 across three datasets (range: +1.209 to +2.096). In contrast, PLIN4 and PRNP showed expression changes opposite to those observed in the mouse qRT-PCR analysis, with mean human log2FC values of +0.452 and −0.452, respectively. Overall, four of six candidate genes showed directionally concordant expression patterns between the mouse findings and the reported human AD transcriptomic results, providing partial cross-species support for our findings. These results also indicate that not all transcriptional alterations identified in the mouse models are conserved in human AD brain tissue.

### 3.8 β-amyloid plaques in the early stage of APP/PS1 mice

**Figure 9:**
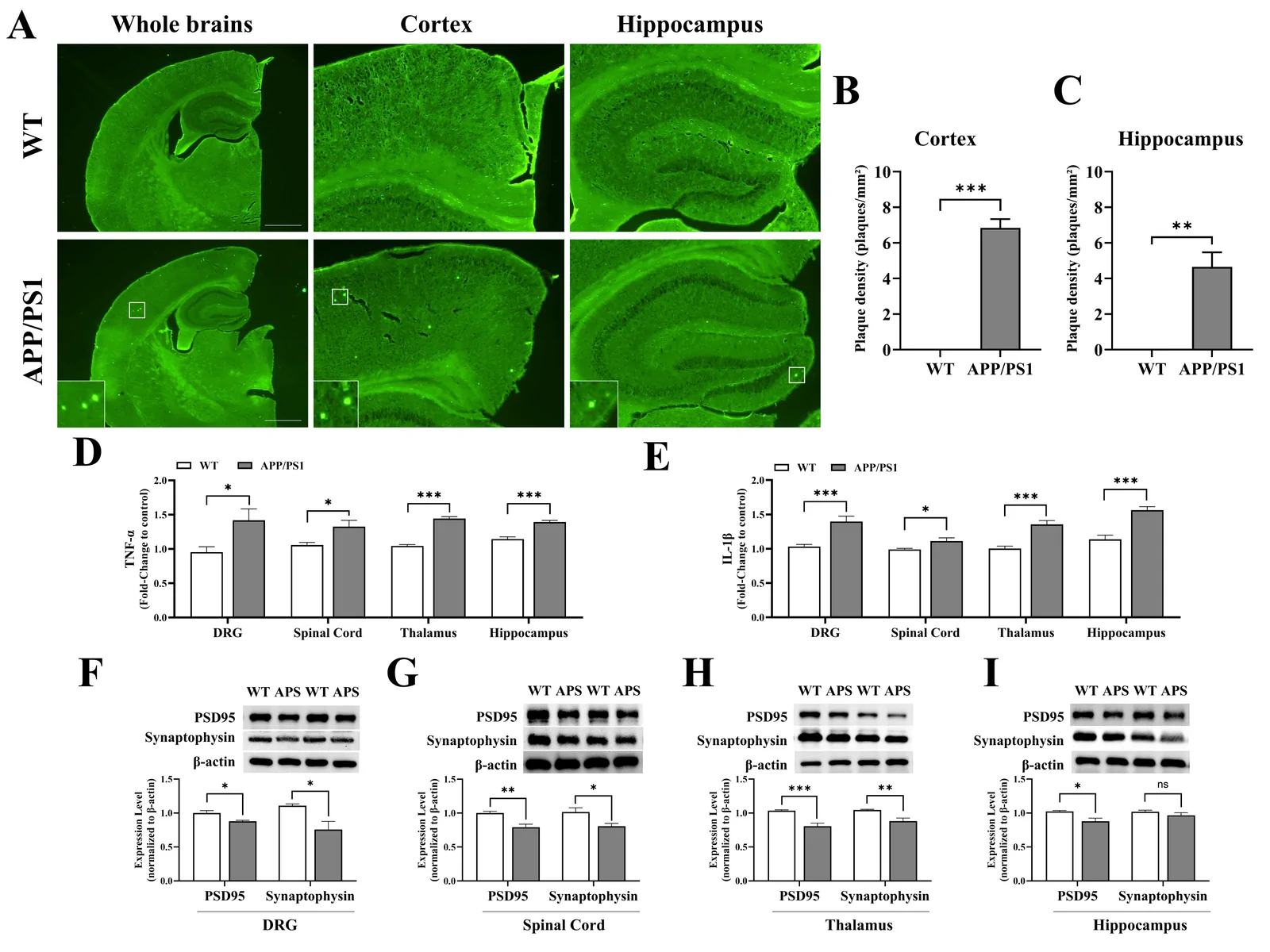
Representative Thioflavin-S staining, cytokine measurements, and synaptic protein analysis in 4–5-month-old APP/PS1 and WT mice. **(A)** Thioflavin S histochemical staining of whole brain, cortex, and hippocampus sections showing Aβ plaques (green) in WT and APP/PS1 mice. Aβ plaque burden was quantified by plaque density (number of Thioflavin S-positive plaques per mm^2^). The boxed region indicates the area shown in the locally enlarged inset. Images are representative of n = 6 brains per group. Scale bar: 500 μm. **(B, C)** Quantification of Aβ plaque number per cross-section in cortex **(B)** and hippocampus **(C)**. **(D, E)** TNF-α **(D)** and IL-1β **(E)** levels, and synaptic proteins expression in the DRG, spinal cord, thalamus, and hippocampus of WT and APP/PS1 mice. **(F–I)** Representative Western blots and corresponding densitometric quantification of PSD95 and synaptophysin in DRG **(F)**, spinal cord **(G)**, thalamus **(H)**, and hippocampus **(I)**. Protein signals were normalized to the β-actin loading control and were presented as relative intensity. Statistical significance was determined by an unpaired t-test (*P < 0.05, **P < 0.01, ***P < 0.001; ns, not significant, unpaired t-test). Data are presented as mean ± SEM. (N = 6). Note: “APS” indicates “APP/PS1”.

The accumulation of amyloid-β (Aβ) into plaques in the AD brain was a hallmark feature of AD pathology. In APP/PS1 mice, Aβ plaques typically emerged in the brain around 4–5 months old. To assess early plaque formation, we performed Thioflavin S staining for Aβ in the brains of APP/PS1 and WT mice at 4–5 months old. As seen in **Figure 9A**, APP/PS1 mice showed scattered Aβ plaques in the cortex, and to a lesser extent, in the hippocampus, whereas WT controls did not display Aβ plaques in the brain regions. Quantitative analysis revealed a significant increase in the number of Aβ plaques per cross-section in both the cortex (**Figure 9B**) and hippocampus (**Figure 9C**) of APP/PS1 mice compared to WT. These findings confirmed the early presence of Aβ pathology in the cortex and hippocampus in this AD model. Notably, despite the presence of Aβ plaques in the cortex and hippocampus, we did not observe any Aβ plaque deposition in the dorsal root ganglion (DRG) or spinal cord at this early stage (data not shown). However, this does not exclude the possibility that soluble Aβ species may exert local effects in the DRG and spinal cord. Future studies directly measuring soluble Aβ1–42 in these tissues will be important to further refine this interpretation. This suggests that molecular alterations observed in pain-processing regions may occur independently of local Aβ plaque deposition. Early nociceptive hypersensitivity in this AD model may be driven by mechanisms independent of local Aβ accumulation in sensory pathways and highlights the complexity of the relationship between amyloid pathology and sensory processing in AD.

### 3.9 Neuroinflammatory and synaptic proteins in the early stage of APP/PS1 mice

To investigate region-specific neuroinflammatory and synaptic changes associated with nociceptive response associated with AD progression, we focused on the DRG, spinal cord, thalamus, and hippocampus, the anatomically and functionally distinct regions linked to AD pathogenesis and nociceptive function. TNF-α levels were considerably higher in the DRG, spinal cord, thalamus, and hippocampus of 4-5-month-old APP/PS1 compared with age-matched WT (**Figure 9D**). On the other hand, APP/PS1 mice showed significantly greater levels of IL-1β in the DRG, spinal cord, thalamus, and hippocampus (**Figure 9E**), suggesting that inflammatory signaling pathways were widely activated. Significant changes in the expression of synaptic proteins were found by Western blot analysis. In the DRG, APP/PS1 mice showed reduced PSD95 and Synaptophysin levels compared with WT mice (**Figure 9F**). A similar decrease was observed in the spinal cord (**Figure 9G**) and thalamus (**Figure 9H**), where both PSD95 and Synaptophysin were significantly lower in APP/PS1 mice. In the hippocampus, PSD95 was decreased in APP/PS1 mice, while synaptophysin showed no significant difference compared with WT controls (**Figure 9I**). These findings demonstrate region-specific synaptic alterations in APP/PS1 mice. The increased levels of inflammatory factors, such as TNF-α and IL-1β, combined with decreased synaptic proteins synaptophysin and PSD95 in 4-5-month-old APP/PS1 mice, suggest that neuroinflammation and synaptic dysfunction are early pathological features in these regions during AD progression.

## 4 Discussion

Comprehensive behavioral characterization showed that APP/PS1 mice at 4–5 months of age had intact cognitive performance but altered nociception. Specifically, APP/PS1 mice at 4–5 months did not exhibit changes in learning and memory performance in either the novel object recognition or Y-maze tests. In contrast, the APP/PS1 cohort showed mechanical hypersensitivity and thermal hyperalgesia. Separate testing at 2 months confirmed that these nociceptive changes are not present earlier, including the mechanical and thermal hypersensitivities. These behavioral results support selecting 4–5 months for our study on nociception alteration.

Transcriptomic analyses of the hippocampus, thalamus, DRG, and spinal cord regions of these mice identified six genes that were altered. In general, the gray matter loss and disruptions in neural circuit connectivity may reduce pain sensitivity in late-stage AD patients (43, 44). At the same time, pain assessment in AD and related dementia (ADRD) may also become compromised over time, primarily due to memory and cognitive deficits (45, 46). Back in 1999, Benedetti and his team reported that pain threshold and tolerance in patients with AD change progressively with disease development (43). They found decreased pain sensitivity in the early stage of AD, but increased pain tolerance in the later stage. Therefore, the present study primarily focused on molecular alterations in pain-processing regions in early-stage AD mouse models. Some supraspinal brain regions are positively associated with pain sensitivity, such as the thalamus and hippocampus (47). In addition to supporting problem-solving and cognitive functions, frontal and related subcortical regions, such as the cingulate cortex, insular cortex, and thalamus, also serve as emotional regulators, influencing processes including depression, anxiety, and pain perception (48, 49). Increasing evidence suggests that both the thalamus and hippocampus are involved in the development of emotional changes, and that the hippocampus-to-thalamus circuit plays a critical role in the expression of pain behavior (50). During the development of AD, multiple pathological pathways within the central nervous system (CNS), e.g., the thalamus, hippocampus, and spinal dorsal horn, as well as peripheral nociceptors, including dorsal root ganglia, coordinate to integrate nociceptive signaling, potentially leading to imbalances in communication networks. The neurons in the supraspinal brain regions regulate the balance between pro-inflammatory and anti-inflammatory processes, and disruptions in this balance can be observed as early as the initial stages of AD pathology (51). The regional selection in the present study was designed to capture anatomically distinct components of the nociceptive pathway, including the DRG, spinal cord, and thalamus, together with the hippocampus as a key region involved in cognition and AD pathology. The thalamus was prioritized over the cortex as the supraspinal region because it serves as a major relay and integrative hub for ascending nociceptive signals and participates in cognitive and limbic networks (52). Pathological dysregulation in the brain may contribute to altered pain processing; however, whether this leads to sensitization of peripheral nociceptors in the DRG remains to be determined (53). However, how early molecular alterations contribute to the late-onset cognitive consequences in individuals carrying AD-related genetic predispositions remains unclear. qRT-PCR validation confirmed region- and gene-specific expression changes identified by RNA-seq analysis. qRT-PCR confirmed that *Plin4* was downregulated in the DRG, spinal cord, thalamus, and hippocampus. *Hif3a* and *Serpina3f* were upregulated in the thalamus and hippocampus. Furthermore, *Prnp* and *Serpina3f* were upregulated in the spinal cord and thalamus. Although marked differences in DEG numbers across regions were unexpected, the transcriptional responses to early AD pathology appeared highly region dependent. These distinct sets of genes may act in a coordinated manner to drive pathological changes during the early stages of disease progression. Further GO analysis revealed that these genes may be involved in neuroinflammatory processes. For example, *Plin4* is mainly downregulated in the above regions, which are implicated in lipid droplet generation and cellular stress responses, and its dysregulation may increase susceptibility to neuroinflammatory injury (54, 55). *Prnp* is related to oxidative stress and the regulation of protease activity (56). Additionally, *Hmga1b*, which was upregulated in the spinal cord, plays a role in transcriptional regulation and chromatin remodeling in response to inflammation and oxidative stress (57). Although the six genes identified here represent the only statistically significant DEGs shared across all four regions under our predefined thresholds, additional genes may exhibit consistent directional trends without reaching statistical significance and therefore warrant consideration in future validation studies.

Multiple studies have implicated these genes in neuroinflammation and neurodegeneration. In particular, dysregulated *Plin4* expression has been observed in the spinal cord of SOD1*G93A transgenic mice during disease progression, suggesting an association with neurodegenerative pathology (58). Studies on *Plin4* gene in peripheral rotator cuff injury suggest that the RNA levels of *Plin4* are downregulated in skeletal muscle, implicating its essential role in oxidative stress and metabolic dysfunction, particularly affecting muscle contraction and shoulder joint pain perception (59). *Hmga1b*, a member of the HMGA protein family, has been linked to the regulation of essential inflammatory pathways, including TNF-α/NF-κB, and plays an essential role in chromatin remodeling and gene transcription, suggesting its potential involvement in neuroinflammatory responses (60). Other studies have found that *Prnp*, the gene encoding prion protein, is upregulated in response to environmental stressors, with strong links to endoplasmic reticulum stress and inflammation (61, 62). The cellular prion protein, encoded by the *Prnp* gene, binds beta-amyloid (Aβ) oligomers with high affinity at the neuronal surface (63). This has been identified in both mouse models and human AD brains (64–66). However, a previous study found that prion protein null mice showed reduced nociceptive thresholds in models of inflammatory pain but not in models of spontaneous pain (67, 68). Collectively, the altered expression of these genes in specific neural compartments highlights their individual contributions to neuroinflammatory processes associated with AD.

Based on these findings, we measured the levels of TNF-α and IL-1β in selected anatomically and functionally distinct regions, the DRG, spinal cord, thalamus, and hippocampus, which represent key regions involved in AD pathology and nociceptive processing. Increased IL-1β and TNF-α levels, together with reduced PSD95 and synaptophysin expression, were detected in pain-related tissues, suggesting that inflammatory activation and synaptic alterations coexist in APP/PS1 mice at this stage. The cytokine elevation may lead to a more severe pro-inflammatory response in 4–5-month-old APP/PS1 mice. We also evaluated the expression of PSD95 and synaptophysin, postsynaptic and presynaptic proteins involved in the development and maintenance of chronic pain and central sensitization (69). We found significant decreases in these two proteins in the DRG, spinal cord, and thalamus. In the hippocampus, the selective reduction in PSD95, while synaptophysin remained unchanged in APP/PS1 mice, suggests that postsynaptic dysfunction may precede presynaptic degeneration in this region. The pattern suggests that neuroinflammation may contribute to synaptic protein loss, providing a mechanistic link between cytokine elevation and synaptic pathology (70). Across distinct regions indicates that AD pathology related to early synaptic dysfunction is accompanied by increased inflammation involved in pain modulation. Interestingly, further observations revealed a dispersed distribution of β-amyloid plaques in the cortex and hippocampus, but none were found in the DRG or spinal cord. Because soluble Aβ oligomers were not measured in the DRG or spinal cord, we cannot exclude the possibility that soluble Aβ contributed to local molecular alterations despite the absence of detectable plaques. Although amyloid pathology may contribute to inflammatory responses, neuroinflammation can also occur independently of detectable plaque accumulation and may reflect broader disease-related processes. These findings provide evidence to support the hypothesis that neuroinflammation occurs in conjunction with β-amyloid buildup and may be a factor in the development of pain perception prior to the manifestation of memory impairments. The evidence that Alzheimer’s disease shows chronic immune dysregulation rather than purely amyloid drives the pathology (71, 72). The activation of microglia and astrocytes, along with blood-brain barrier dysfunction, can initiate inflammatory pathways that affect neural signaling involved in pain processing before cognitive dysfunction emerges (72, 73). Therefore, altered nociceptive processing may represent a potential non-cognitive indicator of early AD-related pathological changes before memory deficits become evident (74). These findings indicate that altered nociceptive perception may serve as an accessible, readily noticeable marker for preclinical detection of AD (5, 75). These findings indicate early transcriptomic alterations in the APP/PS1 mice at this pathological stage.

**Figure 10:**
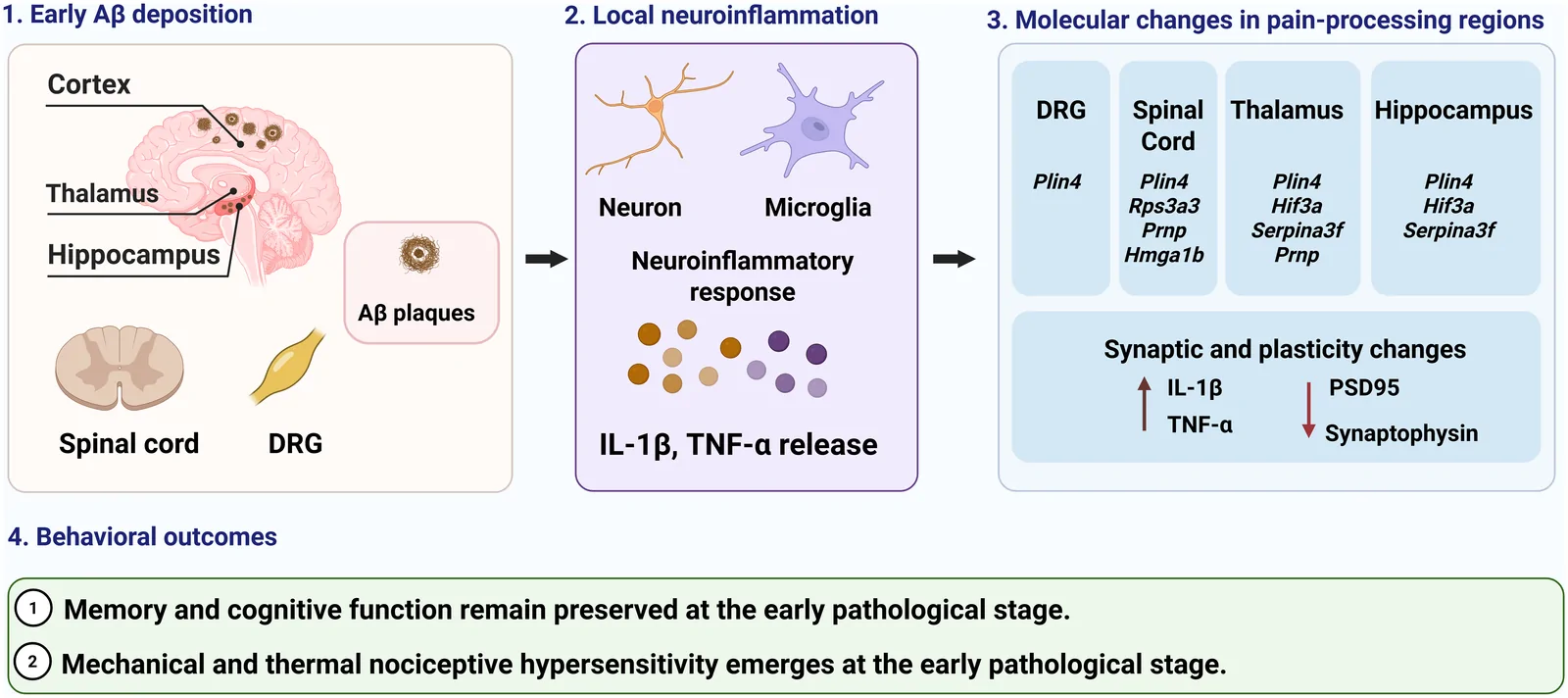
Schematic summary of the study findings in APP/PS1 mice. The diagram presents a conceptual framework linking early Aβ pathology with nociceptive hypersensitivity based on the observed data (Created with BioRender).

In brief, the present study’s findings suggest a possible link between the early pathological stage of Aβ pathology and nociceptive alterations in APP/PS1 mice (**Figure 10**). Early Aβ deposition in the cortex and hippocampus may trigger local glial activation and cytokine release, which could potentially propagate to the spinal cord and DRG. Within pain-processing regions, downregulation of *Plin4* in DRG, spinal cord, thalamus, and hippocampus may contribute to disrupted lipid droplet homeostasis, while spinal cord and thalamus upregulation of *Prnp* could reflect an amplification of inflammatory transcriptional programs. In the thalamus and hippocampus, upregulation of the *Hif3a* and *Serpina3f* could reflect hypoxia response and the inhibition of serine proteases. These molecular changes may partially drive the observed reductions in synaptic proteins PSD95 and synaptophysin, the observed elevations in IL-1β and TNF-α, and the behavioral nociceptive hypersensitivity detected at 4–5 months but not at 2 months of age. The proposed model remains to be tested in future mechanistic studies. Notably, cognitive circuits appeared functionally preserved at this stage, suggesting that sensory alterations may precede cognitive decline in this model.

Functional enrichment analysis of the six shared genes identified potential associations with Ferroptosis and PPAR signaling, along with neuronal, synaptic, protease-regulatory, and lipid-related functions. However, given the relatively modest adjusted significance of the enriched pathways, these findings should be considered hypothesis-generating rather than definitive evidence of pathway activation or mechanistic involvement. Further experimental validation will be necessary to determine whether these pathways directly contribute to the observed molecular and nociceptive phenotypes. Importantly, comparison with publicly available human AD transcriptomic results reported provided partial cross-species support, with four of six candidate genes (*HIF3A*, *HMGA1*, *RPS3A*, and *SERPINA3*) showing concordant expression directions, whereas *PLIN4* and *PRNP* were discordant. Notably, *SERPINA3* showed the strongest and most consistent increase across the human datasets. These findings suggest partial conservation of the transcriptional changes identified in our mouse models, while highlighting potential species-, disease-, and region-specific differences in human AD.

While this study provides multi-regional transcriptomic evidence of early neuroinflammation in APP/PS1 mice, several limitations should be noted. First, we used bulk RNA-seq on pooled tissue samples, with each group consisting of three biological replicates derived from pooled brains. This assay limits the ability to resolve cell-type-specific effects, and the contributions of microglia, astrocytes, neurons, and other cell populations to the observed transcriptomic changes remain to be determined. A single-cell RNA-seq approach would provide greater resolution to identify the specific cell populations contributing to these changes. Second, molecular validation focused on a subset of candidate genes and regions, and protein assays were limited. The functional enrichment analysis based on a small overlapping gene set was inherently limited in statistical power, and enrichment terms with only modest adjusted significance should not be interpreted as definitive mechanistic evidence. Accordingly, the present findings were now presented as potential biological trends that require further validation. Addressing these limitations in future work will strengthen the mechanistic and translational impact of the observed region-specific transcriptomic signatures. Finally, we note that untargeted metabolomics data were acquired from the same cohorts but are not analyzed here. Future integration of metabolomic and transcriptomic datasets, along with targeted biochemical validation, will be essential to clarify metabolic contributions to the observed neuroinflammatory and synaptic changes and to strengthen mechanistic and translational conclusions.

## 5 Conclusions

In conclusion, this study revealed that early-stage neuroinflammation and nociceptive hypersensitivity precede the development of memory impairments and significant β-amyloid formation in 4–5-month-old APP/PS1 mice. The identification of genes provides molecular insights into the altered nociceptive processing pathway and offers a potential molecular signature for early-stage AD progression. These early pathological-stage molecular changes in pain-related regions, occurring before overt cognitive decline in 4-5-month APP/PS1 mice, suggest that nociceptive alterations may serve as an early, accessible indicator of underlying AD pathology. Future studies integrating metabolomics data and targeted protein validation are needed to establish the mechanistic and translational significance of these findings.

## Data Availability

The datasets presented in this study can be found in online repositories (https://doi.org/10.5281/zenodo.17517310). The names of the repository/repositories and accession number(s) can be found in the article/**Supplementary Material**.

## Supplementary material

Supplementary Table 1 Sequencing data statistics.

Supplementary Table 2 Region-specific filtered DEGs (APP/PS1 vs WT). Dorsal root ganglion differential expression analysis between APP/PS1 and WT samples; Hippocampus differential expression analysis between APP/PS1 and WT samples; Spinal cord differential expression analysis between APP/PS1 and WT samples; Thalamus differential expression analysis between APP/PS1 and WT samples.

Supplementary Table 3 Region-specific filtered DEGs (APP/PS1 vs WT). Dorsal root ganglion differential expression analysis between APP/PS1 and WT samples; Hippocampus differential expression analysis between APP/PS1 and WT samples; Spinal cord differential expression analysis between APP/PS1 and WT samples; Thalamus differential expression analysis between APP/PS1 and WT samples.

Supplementary Table 4 Six genes, Plin4, Hif3a, Rps3a3, Serpina3f, Hmga1b, and Prnp, GO and KEGG Pathway Enrichment Analysis between APP/PS1 and WT samples.

Supplementary Table 5 Human AD Dataset Cross-Validation.
